# Highly suitable LFBK cells for African swine fever virus replication and type I interferon-induced immune studies

**DOI:** 10.1186/s13567-025-01543-7

**Published:** 2025-06-11

**Authors:** Eun-Gyeong Lee, Sang-Min Kang, Dongseob Tark

**Affiliations:** 1 Laboratory for Infectious Disease Prevention, Korea Zoonosis Research Institute, Jeonbuk National University, Iksan, 54531 Republic of Korea; 2 ViEL-T Corporate Research Institute, ViEL-T lnc., Jeonju Innovation Startup Hub SJ Bldg, Jeonju, 54852 Republic of Korea

**Keywords:** ASFV, LFBK, PAMs, Type I IFN, suitable cell line

## Abstract

African swine fever virus (ASFV), the causative agent of African swine fever, is a fatal haemorrhagic virus affecting domestic pigs and wild boars. The primary target cells for ASFV infection are porcine alveolar macrophages (PAMs); however, PAM isolation and maintenance are technically challenging, and genetic manipulation of these cells is difficult. The lack of suitable cell lines that support ASFV infection and replication has significantly hindered vaccine development. This study aimed to overcome these limitations and advance ASFV research. The results demonstrate that the foetal porcine kidney cells (LFBK) are suitable for ASFV studies. We observed that ASFV replicated significantly more efficiently in LFBK cells than in PAMs. Furthermore, LFBK cells exhibited antiviral immune responses similar to PAMs following ASFV infection or DNA analog. These findings suggest that the LFBK cell line could provide a much-needed platform for studying ASFV replication and pathogenesis while serving as a valuable tool for understanding ASFV-induced immune mechanisms.

**Supplementary Information:**

The online version contains supplementary material available at 10.1186/s13567-025-01543-7.

## Introduction

African swine fever virus (ASFV) is the only member of the *Asfarviridae* family and the causative agent of African swine fever (ASF), a highly contagious haemorrhagic disease affecting domestic pigs and wild boars, with mortality rates of approximately 100% [1]. ASFV is a large, double-stranded DNA virus with an icosahedral capsid that displays an average diameter of 200 nm. Its genome ranges between 170–190 kilobases in length and encodes more than 150 proteins. In recent years, ASF has spread rapidly across Europe and Asia, with no effective preventive vaccine available to date. Outbreak control has, therefore, relied primarily on quarantine measures and culling infected or exposed animals, resulting in substantial economic losses in the swine industry and posing a global threat [2, 3].

ASFV exhibits cellular tropism of the myeloid lineage, particularly macrophages and monocytes [4]. Viruses are typically isolated from porcine macrophages; however, this approach is time-consuming, labour-intensive, and costly, as the required cells must be harvested from porcine blood or lungs [5]. Such an approach also poses ethical concerns and complicates applications in diagnostic laboratories. Moreover, ASFV reportedly replicates inefficiently and only upon adaptation through sequential passages in specific cell lines. However, suitable cell lines for ASFV isolation and propagation are lacking [6–8]. The limited understanding of the innate immune mechanisms against ASFV has also hindered effective vaccine development [9]. Interferons (IFNs) are pivotal for antiviral defence, particularly type I IFNs (IFN-α/β), which are the most antiviral cytokines for innate immunity against viral infections [10]. The induction of IFN-β requires the corresponding activation of the IFN-stimulated response element (ISRE) and NF-κB [11].

In this study, we identified a suitable cell line for ASFV propagation and studied its innate immunity response against ASFV infection. This cell line, LFBK, is derived from foetal porcine kidney cells and is known to be highly susceptible to foot-and-mouth disease virus infection [12, 13]. However, this cell line’s potential has not yet been explored for its ASFV replication and related antiviral immune responses. Here, we evaluated the viral replication capacity and the related viability in ASFV-infected LFBK cells and compared their ASFV-induced antiviral immune responses with those of LFBK cells and primary porcine macrophages. Additionally, due to the lack of porcine-specific antibodies for detecting antiviral immune mechanisms, we used HEK293 cells to compare protein expression levels in LFBK cells.

The ultimate aim of this study was to determine whether LFBK cells represent a viable cell line for ASFV studies and to establish a foundation for future research into ASFV protein functions.

## Materials and methods

### Chemicals and reagents

Dulbecco’s modified Eagle medium (DMEM), Roswell Park Memorial Institute (RPMI) 1640 Medium, foetal bovine serum (FBS), phosphate-buffered saline (PBS; pH 7.4), penicillin (100 U/mL), streptomycin (100 μg/mL), Lipofectamine 2000 transfection reagent, and the VetMAX African Swine Fever Virus Detection Kit were purchased from Thermo Fisher Scientific (Waltham, MA, USA). The CellTiter-Glo Luminescent Cell Viability Assay Kit, Bright-Glo Luciferase Assay System, and Beta-Glo Assay System were obtained from Promega (Madison, WI, USA). Poly (dA:dT) and poly(dA:dT)/Lyovec were purchased from InvivoGen (San Diego, CA, USA), and jetOPTIMUS DNA transfection reagent were sourced from Polyplus (Illkirch-Graffenstaden, France).

Rabbit anti-phospho-IRF3(Ser396), IRF3, phospho-STAT1(Ser727), STAT1, phospho-NF-κB p65, NF-κB p65, phospho-IκBα(Ser32/36), IκBα, PKR, ISG56, mouse anti-β-actin, goat anti-rabbit IgG-HRP, goat anti-mouse IgG-HRP, and Alexa Fluor 488-conjugated goat anti-mouse IgG antibodies were purchased from Cell Signaling Technology (Boston, MA, USA). The MX1 antibody was acquired from Abcam (Cambridge, UK), and the mouse anti-ASFV p72 antibody was purchased from Creative Biolabs (Shirley, NY, USA).

### Cells and virus

LFBK (MTA#18926, USDA) and HEK293 (provided by Dr.Kang, JBNU) cells were cultured in DMEM with 10% FBS supplemented with 10 000 U/mL penicillin and 10 000 μg/mL streptomycin (Gibco) at 37℃ and under 5% CO_2_ in an incubator. Primary porcine alveolar macrophages (PAMs) were isolated as previously described [14]. Briefly, PAMs were collected from the bronchoalveolar of specific pathogen-free pigs, then maintained in an RPMI 1640 medium containing 10% FBS and 1% of antibiotics (10 000 U/mL penicillin, 10 mg/mL streptomycin) and antimycotics 25 μg/mL amphotericin B (Sigma-Aldrich, St. Louis, MO, USA) at 37℃ and under 5% CO_2_ in an incubator.

In the present study, the ASFV isolate (Korea/Pig/Paju1/2019, KVCC, VR2000003) was used, amplifying the genotype II strain in PAM cells, incubated at 37 ℃ under 5% CO_2_ for 5–7 days. Following centrifugation at 1000 × *g* for 5 min, the viral stocks were stored at −80 ℃. For PAMs, viral titres were determined using the 50% haemadsorption (HAD_50_) dose method; the CD2v protein mediates ASFV haemadsorption around infected porcine macrophages in the presence of porcine red blood cells, and LFBKs were calculated using a 50% tissue culture infectious dose (TCID_50_).

PAM cells were seeded in 96-well plates, and the viruses were added to the plates and titrated in triplicate tenfold serial dilutions. After 1 h, the medium was replaced to include 2% porcine red blood cells (RBC), and the haemadsorption assay was performed on day 5 post-inoculation. Virus stocks (1 × 10^6^ HAD_50_/mL) were passaged three times on LFBK cells. The virus titers were determined in triplicate tenfold serial dilutions (range: 1 × 10^−8^–1 × 10^−1^), and cytopathic effect (CPE) was monitored at 3–5 days post-infection (dpi). The HAD_50_ and TCID_50_ values were calculated using the Reed and Muench method [15]. We performed the ASFV infection experiments at a biosafety level 3 laboratory in accordance with the biosafety manual instructions issued by the Korea Zoonosis Research Institute of Jeonbuk National University.

### ASFV RNA copy number determination in cells and supernatants

LFBK cells were seeded onto 6-well plates (4 × 10^5^/well), incubated for 16 h, and then the medium was replaced with serum-free DMEM. The cells were infected at a multiplicity of infection (MOI) of 0.1 (1 × 10^7^ TCID_50_/mL) for 1 h. The supernatant was removed, and the cells were washed thrice with an FBS-free medium. The medium containing 5% FBS was replaced, and the infected cells were incubated for 24, 48, and 72 h at 37℃ under 5% CO_2_. The culture supernatants were collected at the indicated times and the ASFV was isolated from the supernatants by centrifuge for 10 min at 3000 rpm. The LFBK cells were re-infected with isolated ASFV in the 6-well plates. At each indicated time point, the supernatant was collected and cleared by centrifugation for 10 min at 3000 rpm. The LFBK cells were re-infected with the ASFV-containing supernatant in the 6-well plates. After 1 h, The cells were washed with an FBS-free medium to remove the virus and replaced with a culture medium containing 5% FBS. The infected cells were again incubated for 24, 48, and 72 h at 37℃ under 5% CO_2._ The cell culture supernatant was harvested and clarified by centrifugation for 10 min at 3000 rpm to remove the cell debris. The cells were then washed thrice with PBS and collected, and both the cells and the supernatant were frozen at −80℃ at respective times. The viral RNA level was quantified at each time point of the cell and supernatant samples using a VetMAX African Swine Fever Virus Detection Kit, which detects the ASFV-capsid gene *p72* using quantitative reverse transcription polymerase chain reaction (qRT-PCR).

### RNA isolation and reverse transcription real-time quantitative PCR

LFBK and PAM cells were seeded (4 × 10^5^ and 2 × 10^6^/well, respectively) onto 6-well plates, incubated for 16 h, then the medium was replaced with a 5% FBS-containing culture medium. The cells were infected with ASFV at an MOI of 0.1 for the indicated time points. Based on the stock solution preparation method provided by the manufacturer (tlrl-patc, InvivoGen, USA), the poly e(dA:dT)/Lyovec mixture was prepared at a concentration of 10 μg/mL and transfected into LFBK cells for the indicated durations. The supernatants were then removed, and the cells were washed twice with PBS. Total RNA was extracted from the samples using TRIzol reagent (Invitrogen, Waltham, MA, USA) in accordance with the manufacturer’s instructions. The RNA levels were quantified at 100 ng/μL and synthesised cDNA using the CellScript cDNA Master Mix (Cellsafe, Yongin, South Korea).

The PCR reactions were performed as per the manufacturer’s protocol. The ASFV *p72* gene copy levels were quantified using the VetMAX African Swine Fever Virus Detection Kit, which included a positive control. qRT-PCR was performed using the synthesised cDNA as a template under the following thermal conditions: 95 ℃ for 10 s and 58 ℃ for 30 s (for IFN-β, IFN-α, MX1, ISG56, ISG15, and β-actin) or 55 ℃ for 30 s (for IRF7, IRF3, and PKR) for a total of 40 cycles using iQ SYBR Green Supermix (Bio-Rad, Hercules, CA, USA). Amplification was performed using the Quantstudio 3 Real-time PCR system (Thermo Fisher Scientific). The β-actin gene was used as an internal control. Relative mRNA expression levels were calculated using the 2_¯_^ΔΔCT^ method. The primers used in this study are listed in Table 1.

**Table 1:** **qRT-PCR Primer sequences**

Gene	Sequence (5′−3′)	References (NCBI)
β-actin-F	ACCACTGGCATTGTCATGGACTCT	[37]
β-actin-R	ATCTTCATGAGGTAGTCGGTCAGG	
IFN-α-F	ACTCCATCCTGGCTGTGAGGAAAT	[37]
IFN-α-R	TCTGTCTTGCAGGTTTGTGGAGGA	
IFN-β-F	CACTGGCTGGAATGAAACCG	[38]
IFN-β-R	AATGGTCATGTCTCCCCTGG	
ISG15-F	GGTGCAAAGCTTCAGAGACC	[22]
ISG15-R	GTCAGCCAGACCTCATAGGC	
ISG56-F	CCCACTTCTGTCTTACTGC	[22]
ISG56-R	TACATTCTTGCCAGGTCTA	
MX1-F	CACCTGAAGAAGGGCTACATGAT	(DQ095779)
MX1-R	AACAGGGGCAGAGTTTTACAGAT	
IRF7-F	ATCTGCTGGAAGCTGCAT	(100,037,289)
IRF7-R	ACATGATGGTCAAGTCCAGG	
IRF3-F	CCACGAAGACAGTCTGGATAA	(396,656)
IRF3-R	TAGAAGACGGTCACCTGGAA	
PKR-F	CAGAAAGCAGTGATAACAGTGACA	(AB104654)
PKR-R	TTGAAAACTTGGCCAAATCCACC	

IFN-β: interferon-beta; IFN-α: interferon-alpha; ISG15: interferon-stimulated gene 15; ISG56: interferon-stimulated gene 56; MX1: Myxovirus resistance 1; IRF7: interferon regulatory factor 7; IRF3: interferon regulatory factor 3; PKR: protein kinase R.

### Cell viability assay

Cell viability following ASFV infection or stimulation with transfected poly(dA:dT) in LFBK cells was assessed by measuring ATP levels using the Cell Titer-Glo Luminescent Cell Viability Assay Kit. LFBK cells were seeded in 96-well plates (1 × 10^4^/well) and incubated for 16 h. The supernatants were then removed and added to a serum-free DMEM. Cells were inoculated with ASFV at dilutions ranging from 1 × 10^–8^ to 1 × 10^−1^. After 1 h, the diluents were removed, and the medium was replaced with 5% FBS-containing DMEM, followed by incubation for the indicated time points. Next, the LFBK cells were transfected poly(dA:dT)/Lyovec in a dose-dependent manner (1, 3, 10, 20, 30, and 40 μg/mL) for 12, 24, 48, and 72 h. At the respective time points following ASFV infection or poly(dA:dT)/Lyovec transfection, 100 μL of Cell Titer-Glo reagent was added per well. The plate was shaken for 2 min at 700 rpm and then incubated for 10 min. Luminescence was measured using a luminometer. Based on the registered luminescence, the percentage of viable cells was calculated by comparing the infected or transfected wells as a ratio to the mean viability of naïve LFBK cells. All experiments were conducted in triplicates.

### Luciferase reporter assay

LFBK (4 × 10^5^/well) and HEK293 (8 × 10^5^/well) cells were seeded in 6-well plates and incubated for 16 h at 37℃ under 5% CO_2_. The medium was then replaced with DMEM containing 10% FBS, and the cells were co-transfected with 0.05 μg of β-galactosidase plasmid and 0.6 μg of reporter plasmid (either IFN-β or ISRE) in LFBK or the same dose of β-galactosidase plasmid and 0.2 μg of reporter plasmids in HEK293 cells, along with the indicated empty vector using the jetOPTIMUS DNA transfection reagent. An empty vector was included in the transfection assay to ensure the cells received equal amounts of total plasmids. To normalise transfection efficiency, the β-galactosidase plasmid was added to each transfection. At 24 h post-transfection, the medium was replaced with fresh DMEM containing 10% FBS. LFBK cells were then infected with ASFV at an MOI of 0.1 for 24 h or further transfected with poly(dA:dT) at 3 μg/mL (LFBK) or 1 μg/mL (HEK293) using Lipofectamine 2000 (1:2 ratio) for 12 h. Following infection or transfection, luciferase and β-galactosidase assays were performed using a luciferase assay system according to the manufacturer’s instructions.

### Immunoblotting

In this study, we aimed to detect ASFV *p72* protein expression and antiviral immune signalling, depending on the time. LFBK cells were seeded in 6-well plates (4 × 10^5^/well) and incubated for 16 h at 37℃ under 5% CO_2_. The medium was changed with DMEM containing 5% FBS and the cells were infected with ASFV at an MOI of 0.1 for the indicated times. The HEK293 cells were seeded onto 6-well plates (8 × 10^5^/well) and stimulated with transfected poly(dA:dT) (1 μg/mL) at the indicated times using Lipofectamine 2000 (1:2 ratio) and according to the manufacturer’s instructions. The supernatants were removed, and the cells were washed thrice with PBS, then lysed using RIPA lysis buffer supplemented with a protease and phosphatase inhibitor cocktail (Thermo Fisher Scientific).

The lysed cells were centrifuged at 13 000 × *g* for 15 min at 4 ℃. Equal protein amounts from the lysates were mixed with 4 × Laemmli sample buffers containing 2-mercaptoethanol (Bio-Rad). The proteins were denatured at 100 ℃ for 10 min, separated using SDS polyacrylamide gel electrophoresis, and transferred onto a polyvinylidene fluoride (PVDF) membrane using an iBlot Transfer stack (Thermo Fisher Scientific). The membrane was blocked with 5% skim milk in TBS-T buffer for 1 h at 25 ℃, then incubated at 4 ℃ for 16 h with the indicated primary antibody, diluted to 1:1000 in 1% skim milk. Following six washes with TBS-T buffer, the membrane was incubated with horseradish peroxidase-conjugated secondary antibodies (at a dilution of 1:3000) against rabbit or mouse IgG for 90 min at 25 ℃. After another six washes with TBS-T, we detected the protein bands using a chemiluminescence reagent (Cytiva, Marlborough, MA, USA) and the iBright 1500 imaging system (Thermo Fisher Scientific). Densitometry was used to quantify relative expression levels of the target proteins (iBright, Thermo Fisher Scientific).

### Immunofluorescence assay (IFA)

LFBK and PAM cells (2 × 10^5^ and 1 × 10^6^/well, respectively) were seeded onto 35-mm confocal dishes and incubated for 16 h at 37℃ under 5% CO_2_. The medium was then replaced with DMEM containing 5% FBS, and the cells were infected at an MOI of 0.1. After 2 dpi, the supernatant was removed, and the cells were washed thrice with PBS, then fixed with methanol and acetone (1:1 ratio) for 10 min. Following fixation, the cells were washed thrice with PBS and blocked with 5% BSA for 1 h. Next, the fixed cells were incubated with a *p72* monoclonal antibody (diluted at 1:1000) for 2 h at 25 ℃, then washed thrice with PBS. Cells were subsequently incubated with Alexa Fluor 488-conjugated goat anti-mouse IgG (diluted at 1:2000) for 1 h at 25 ℃. After washing thrice with PBS, the nuclei were stained with 4′,6′-diamidino-2-phenylindole (DAPI) (Thermo Fisher Scientific, Waltham, MA, USA) for 10 min. Fluorescent imaging was performed using a confocal laser scanning microscope with a CELENA® S Digital Imaging System (Logos Biosystems, Anyang, South Korea).

### TCID_50_ assay

The ASFV-infected LFBK cells and their supernatants were subjected to three freeze–thaw cycles performed at −80℃. The clarified supernatant was collected by centrifugation for 10 min at 3000 rpm, and then the samples were serially diluted with serum-free DMEM.

The LFBK cells (1 × 10^4^/well) were seeded into 96-well plates and inoculated in quadruplicate with serial dilutions of the virus, ranging from 1 × 10^−8^ to 1 × 10^−1^ for 1 h. Following incubation, the diluents were removed, and the wells were washed thrice with FBS-free DMEM. The samples were then sequentially replaced with DMEM containing 5% FBS, and the CPE was monitored 3 to 5 days after ASFV infection. ASFV TCID_50_ values were calculated using the Reed and Muench method.

### Statistical analysis

Statistical data was analysed using GraphPad Prism 8.0 software (GraphPad Software, San Diego, CA, USA). One-way analysis of variance (ANOVA) was used for multiple comparisons, followed by Tukey’s tests. All experimental results are expressed as the means ± SD, with statistical significance levels set as follows: * p < 0.05; ** p < 0.01; *** p < 0.001; **** p < 0.0001.

## Results

### Determination of ASFV replication and cytotoxicity in LFBK cells

**Figure 1:**
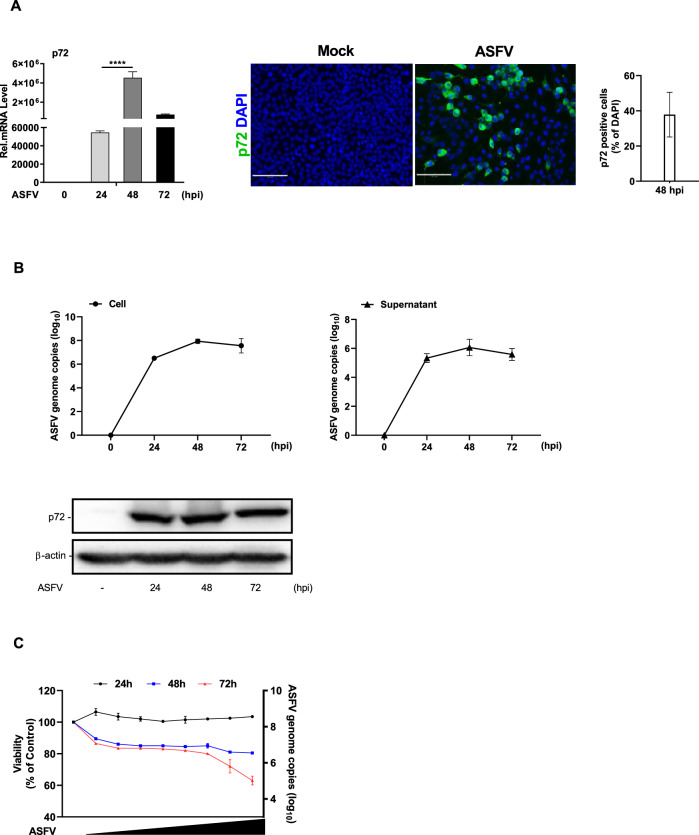
**ASFV propagation determination in LFBK cells. **LFBK cells were non-infected or infected with 1 × 10^7^ TCID_50_ of ASFV at a MOI of 0.1 for the indicated times. **A** ASFV *p72* expression levels were determined by qRT-PCR using the VetMAX African Swine Fever Virus detection kit, and the positive control was presented in the kit. The viral replication state in the LFBK cells was qRT-PCR-quantified. The *p72* gene expression levels were normalised to that of β-actin. Representative immunofluorescence images of the ASFV *p72* protein (green) and DAPI (blue) staining in LFBK cells (48 hpi). Magnification: 100 ×. **B** The cellular and supernatant genomic copy levels of the ASFV *p72* protein were qRT-PCR-quantified at the indicated times for 1 h post-infection in the LFBK cells. Immunoblotting was used to detect the cellular levels of the ASFV *p72* protein at the indicated times after infection. **C** LFBK cell viability was assessed using the Cell Titer-Glo Luminescent Cell Viability Assay. LFBK cells were infected with tenfold serially diluted ASFV for the indicated times. TCID_50_ was used to perform the ASFV titration in the LFBK cells for the infectious virus. The experiments were carried out in triplicates, and the results represent the means ± SD. One-way ANOVA with Tukey’s multiple comparisons test was used to determine the significance level. ****p < 0.0001. dpi: days post-infection.

A 50% tissue culture infectious dose (TCID_50)_ assay was performed to calculate the virus titer of ASFV in LFBK cells. TCID_50_ was determined based on the presence or absence of cytopathic effect (CPE) observed 3 to 5 dpi, and the ASFV TCID_50_ was calculated using the Reed and Muench method (Additional file 1A). To assess the susceptibility of LFBK cells to ASFV infection, cells were infected with ASFV at an MOI of 0.1, and the viral *p72* gene level were quantified using qRT-PCR. The *p72* mRNA expression levels increased significantly up to 48 h post-ASFV infection and remained stable at similar levels after 72 h (Figure 1A). In addition, an immunofluorescence assay revealed that 37.8% ± 12.6% of the total LFBK cells were *p72*-positive. In comparison, *p72*-positive PAMs accounted for only 6.2 ± 1.2% (Additional file 1B). To further assess viral replication, qRT-PCR was performed to quantify the genomic copy number of *p72* in the cells and supernatants, with corresponding *p72* protein levels confirmed in LFBK cells (Figure 1B).

Cell lysates and supernatants were collected from each sample, and the intra- and extracellular ASFV genomic copies were quantified. We observed an increase in the DNA copy numbers between 24–72 hpi in both cell lysates (average: 6.5–7.5 log_10_) and supernatants (average: 5.3–5.5 log_10_), and the *p72* protein level increased at the indicated times. The viability of the ASFV-infected LFBK cell was also examined (Figure 1C). At 24 hpi, cell viability of the LFBK cells was not cytotoxic on the serial diluents (Range: 10^−8^ – 10^−1^) and was not significantly different from non-infected cells. At 48 hpi, cell viability ranged from 89 to 81% ± 2%, and at 72 hpi, it ranged from 86 to 63% ± 5% across the same dilution. These data indicate that LFBK cells support efficient propagation without significant ASFV-related cytotoxicity for 48 h.

### Antiviral immune response evaluation at the mRNA expression level in LFBK cells via DNA analogs or ASFV

**Figure 2:**
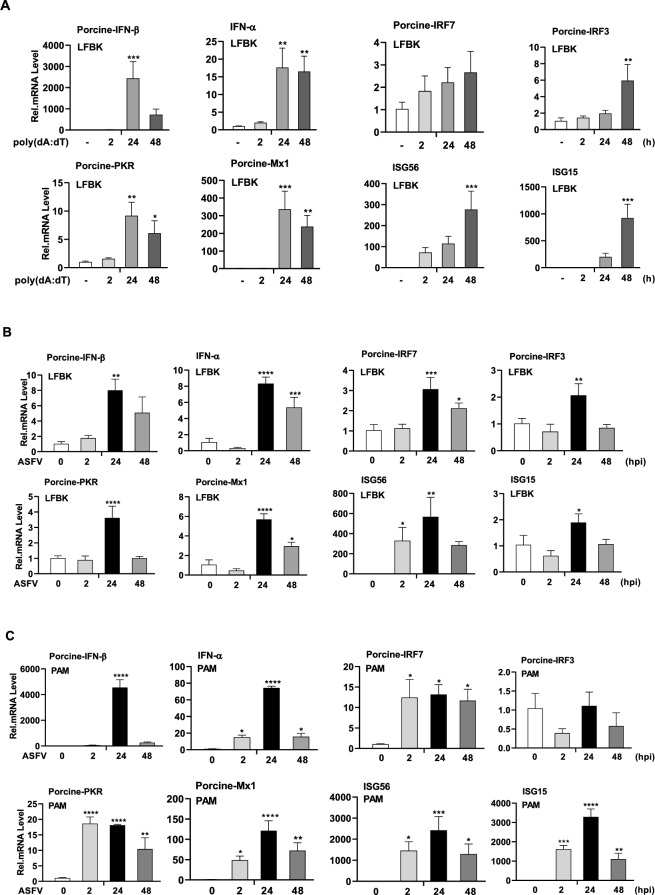
**Evaluation of the antiviral immune response activities in LFBK cells and PAM cells.** The cells were non-infected or infected with ASFV (MOI of 0.1) for the indicated times. **A** To investigate the antiviral immune responses with stimulation of DNA analogs analyzed using qRT-PCR at the indicated times upon stimulation with transfected poly(dA:dT) (10 μg/mL) in LFBK cells. **B**, **C** Porcine INF-β1, IRF7, IRF3, PKR, MX1 and, INF-α, ISG15, and ISG56 mRNA expression levels were analysed using qRT-PCR at the indicated times post-infection in LFBK or PAM cells *Denotes significance compared to the non-infected or non-transfected control groups. The experiments were carried out in triplicates, and the results represent the means ± SD. One-way ANOVA with Tukey’s multiple comparisons test was used to determine the significance level. *p < 0.05; **p < 0.01; ***p < 0.001; ****p < 0.0001.

Antiviral immune responses are the first line of host defence mechanisms against viral infection, activating antiviral effector-related transcription factors and synthesis, including type I interferons (IFNs), interferon regulatory factors, and their downstream interferon-stimulated genes (ISGs) [16]. To investigate innate antiviral responses, LFBK cells were transfected with poly(dA:dT). Time- and dose-dependent cell viability tests were performed to identify poly(dA:dT)/Lyovec-related cytotoxicity in LFBK cells (Additional file 2). LFBK cells stimulated with transfected poly(dA:dT) (10 μg/mL) to detect IFN-β, IFN-α, IRF7, IRF3, PKR, MX1, ISG56, and ISG15 at 2, 24, and 48 h infection times using qRT-PCR was investigated (Figure 2A). Our findings show that the poly(dA:dT) stimulation significantly induces IFN-α, IFN-β, PKR, and MX1 at 24 h, IRF3, ISG56, and ISG15 mRNA levels at 48 h. IRF7 expression, however, was not significantly induced between times in LFBK cells.

To assess whether ASFV induces these antiviral effectors, LFBK cells were infected with ASFV for 2, 24, and 48 h and compared with PAM cells (Figures 2B and C). We found that the ASFV infection significantly induced IFNs (IFN-α and IFN-β), transcription factor (IRF7, IRF3), and ISGs (PKR, MX1, ISG56, and ISG15) in the LFBK cells at 24 hpi. Similar expression profiles were observed in PAM cells, although the mRNA expression levels were higher. These results suggest that LFBK cells can induce a type I IFN response and up-regulate downstream antiviral genes in effect to both DNA analogs and ASFV infection.

### Investigation of the ASFV-induced type I interferon activation and antiviral signalling pathway in LFBK cells

**Figure 3:**
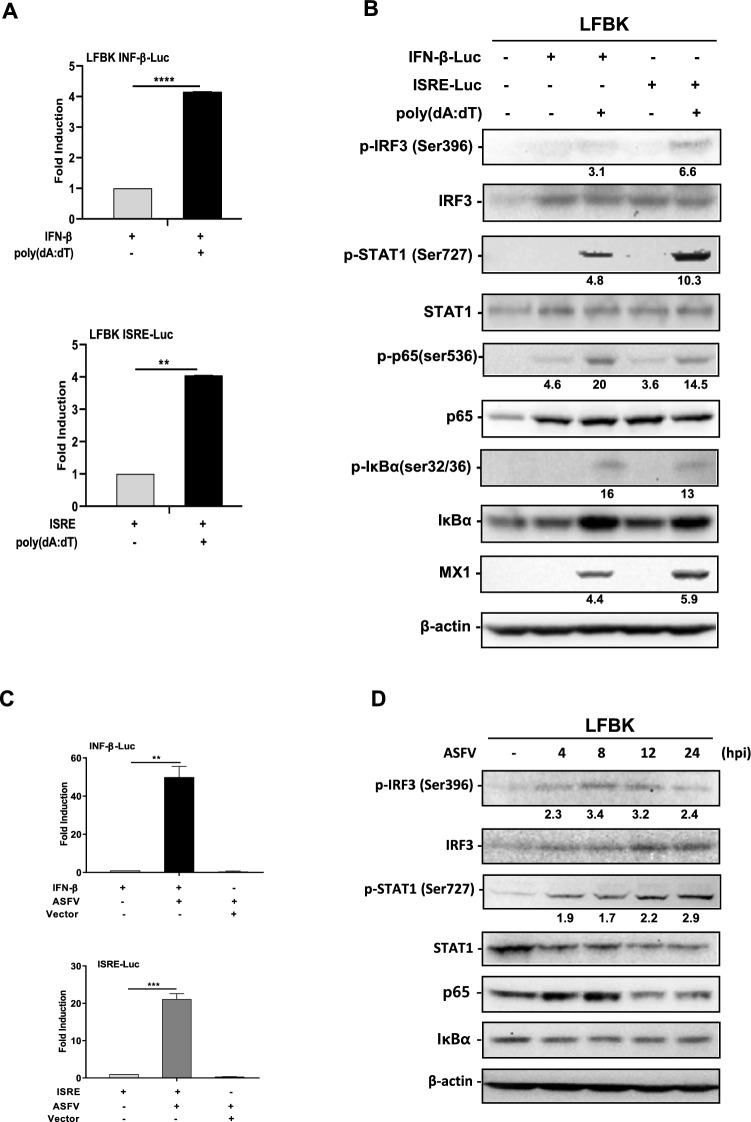
**IFN-β or ISRE promoter activation and the antiviral immune activation in LFBK cells.** **A** LFBK cells were transfected with the IFN-β or ISRE luciferase reporter plasmids and β-galactosidase or empty vectors for 24 h. After 24 h of transfection, the cells were stimulated with transfected poly(dA:dT) (3 μg/mL) for 12 h. A luciferase assay was performed 12 h later. **B** and **D** The cells were lysed and subjected to immunoblot analysis using the indicated antibodies. β-actin was used as a loading control. **C** LFBK cells were transfected with the IFN-β or ISRE luciferase reporter plasmids and β-galactosidase or empty vectors for 24 h. After 24 h of transfection, the cells were left non-infected or were infected with ASFV (MOI of 0.1) for 24 h. 24 h later, a luciferase assay was performed. **D** LFBK cells were infected with ASFV (MOI of 0.1) at the indicated times. The iBright analysis software quantified the band intensities (Invitrogen, Version 5.2.0). The experiments were carried out in triplicates, and the results represent the means ± SD. One-way ANOVA with Tukey’s multiple comparisons test was used to determine the significance level. **p < 0.01; ****p < 0.0001.

To determine whether DNA analogs or ASFV could activate IFN-β and ISRE promoters in LFBK cells, HEK293 cells were used for comparison. Our results demonstrated that HEK293 cells transfected with poly(dA:dT) (1 μg/mL) exhibited induction of IFN-β (37.5 ± 2.3) and ISRE (29.3 ± 2.2) (Additional file 3A). Induction of IFN-β and ISRE gene transcription critically depends on the phosphorylation of key transcription factors, including IRF3, NF-κB, and STAT1. The results showed that phosphorylation of IRF3, STAT1, p65, and IκBα was induced following poly(dA:dT) stimulation in HEK293 cells at 12 h, with a marked increase observed at 24 h. In addition, the ISGs (PKR, MX1, and ISG56) were also increased (Additional file 3B). IFN-β and ISRE promoter activation and immunoblotting were performed to detect the pathway activation following DNA analog stimulation in LFBK cells. The IFN-β (4.1 ± 0.1) and ISRE (4.0 ± 0.8) promoters were activated in LFBK cells following stimulation with poly(dA:dT) at 3 μg/mL (Figure 3A). Furthermore, phosphorylation of IRF3, STAT1, p65, and IκBα was also induced, alongside an increased expression of ISGs, MX1, was observed (Figure 3B).

We sought to determine whether type I IFN signalling functions properly in LFBK cells following ASFV infection. ASFV-induced IFN-β (49.9 ± 1.7) and ISRE (21.1 ± 0.2) reporter activities also were increased in the LFBK cells (Figure 3C). Interestingly, phosphorylation of IRF3 increased between 4 and 8 h post-ASFV infection and gradually decreased thereafter, while phosphorylation of STAT1 continued to rise up to 24 h. Additionally, unlike poly(dA:dT) stimulation, phosphorylation of p65 and IκBα was not induced (Figure 3D). HEK293 and LFBK cells were transfected with IFN-β or ISRE promoter-driven luciferase reporter and internal control empty vectors. These results demonstrate that LFBK cells are suitable for studying the antiviral immune response when to ASFV infection, as well as the virus’s immune evasion mechanisms.

## Discussion

To date, significant research efforts have focused on establishing an in vitro infection model to study ASFV pathogenesis and develop vaccines against this disease. However, no safe and clinically effective vaccines or antiviral therapies are currently unavailable [17, 18]. One major obstacle to a breakthrough is the lack of susceptible cell lines. ASFV primarily replicates in PAMs and porcine blood monocytes, which must be isolated immediately from pigs and cannot be maintained continuously in tissue culture [8, 17, 19]. Therefore, identifying a cell line that allows for stable ASFV replication is crucial for basic research.

Several cell lines have been investigated for ASFV studies [20, 21], including efforts to adapt field isolates to continuous monkey or human cell lines such as Vero and HEK293 T cells. Repeated passages in these lines have been shown to reduce the replication capacity and infectivity in PAMs in vitro [22]. Currently, monkey-derived COS cells and porcine cell lines PK15, WSL, and PIPEC are under investigation as potential alternatives for live attenuated vaccine research. However, further research is needed to investigate the applicability of these cell lines in large-scale vaccine production and to understand any genome mutations that may result in loss of immunogenicity [8]. Additionally, MA-104, a commercially available monkey-derived cell line, could support infection with genotype II ASFV and may provide insights into how ASFV’s evasion of host innate immune responses. However, more analyses would be needed to confirm whether MA-104 is an optimal cell line for ASFV strain propagation [8, 23, 24]. The immortalised PAM-derived porcine alveolar macrophage cell line, particularly the 3D4 variant, and its subclones 3D4/2, 3D4/21, and 3D4/31, is commonly used [25]. However, these cell lines exhibit reduced infection and replication efficiency for several ASFV strains, such as BA71 V, NH/P68, Malawi82, or Lisbon57, and a complete loss of infection capability in others, such as Armenia/07, NH/P68, and ASFV-HLJ/18 [19, 26, 27].

This study evaluated ASFV susceptibility and replication efficiency in the porcine kidney cell-derived LFBK cell line (Figure 1A, B). Using IFA, we confirmed that ASFV replicates more efficiently in LFBK than in PAM cells (Additional file 1B). By confirming ASFV replication in LFBK cells. This finding highlights the need for comprehensive studies on viral pathogenicity, including the characterisation of virulence factors and virus-host interaction mechanisms. We first investigated whether LFBK cells could be used to study the innate immune response to ASFV infection. Following DNA analog stimulation in LFBK cells, we observed increased mRNA expression levels of type I IFNs (IFN-α/β), transcription factors (IRF7/IRF3), and ISGs (PKR, MX1, ISG56, and ISG15) (Figure 2A). These results were comparable to those observed in PAM cells (Figure 2C). Type I IFNs, as the primary cytokines in the innate immune response to early viral infection, have diverse roles in antiviral and immunomodulatory activities [28–31]. Virus-infected cells promote IFN-α and IFN-β production through the nuclear translocation of IRF3, IRF7, and NF-κB. These cytokines bind IFN receptors, leading to phosphorylation and activating the JAK-STAT pathway, which translocates to the nucleus and initiates ISG transcription to combat viral spread [10].

Our findings suggest that LFBK cells are suitable for studying the immune response to ASFV infection. Furthermore, gene knockdown, gene knockout, and gene expression techniques are currently applied to investigate the antiviral effects of host factors during ASFV infection [32, 33]. Using gene expression analysis in LFBK cells, we examined the cellular immune response to ASFV. Our luciferase assays confirmed IFN-β and ISRE promoter activation in LFBK cells following ASFV infection (Figure 3C) or DNA analog stimulation (Figure 3A). ASFV has previously been reported as sensitive to type I IFNs [34]. In LFBK cells, ASFV infection induced IRF3 and STAT1 phosphorylation but did not activate NF-κB and IκB activation upon ASFV infection (Figure 3D). Full activation of NF-κB, IRF3, and AP-1 binding with IFN-β enhancer is needed for high levels of type I IFN expression, contributing to an essential defence against viral infection [35]. Furthermore, NF-κB pathway activation relies on IκBα phosphorylation and degradation, facilitating NF-κB nuclear translocation. A previous study showed that MGF360-12L, one of the multigene family (MGF) of ASFV, impairs the interaction between importin α and NF-κB, thereby inhibiting the nuclear localisation of p65, reducing the host antiviral response activation [36]. In addition, our results indicated that ISG protein expression was not observed following ASFV infection in LFBK cells, whereas DNA analog stimulation in HEK293 did induce ISG expression (Additional file 3B). These findings suggest that ASFV may inhibit NF-κB activation by regulating p65 and IκBα phosphorylation in LFBK cells (Figure 3D). A limitation of this study is the lack of porcine-specific antibodies, which presents challenges for detecting protein expression, emphasising the need to develop such antibodies.

In conclusion, this study identified and characterised the LFBK cell line, indicating the high efficiency and stability of ASFV infection. In this work, the LFBK cells were also determined to be viable for investigating ASFV infection, replication, and the associated innate antiviral immune response. Furthermore, the results presented also support their use in studying intracellular mechanisms through gene knockdown and transfection by conventional laboratory techniques. By utilising this cell line in future studies, we will focus on characterising the numerous ASFV genes with unknown functions.

## Supplementary Information

**Additional file 1: Determination of the ASFV infectivity in LFBK cells or PAMs.** (A) The LFBK cells were infected with 10-fold serially diluted from 1 × 10−8 to 1 × 10−1 diluents of ASFV for 3 to 5 days post-infection (dpi). A 50% tissue culture infectious dose (TCID50) was determined based on whether or not a cytopathic effect occurred. The TCID50 was calculated using the Reed and Muench method. (B) PAM cells were non-infected or infected with ASFV (MOI of 0.1). Representative immunofluorescence images of the ASFV p72 protein (green) and DAPI (blue) staining in PAM cells (48 hpi). The experiments were carried out in triplicates, and the results represent the means ± SD. Magnification: 200×.

**Additional file 2: Cell viability analysis of LFBK cells treated with varying poly(dA:dT) concentrations.** Luminescent cell viability assay was performed at subsequent times after the cells were dose-dependently stimulated with transfected poly(dA:dT) compared to the non-transfected control group. The experiments were carried out in triplicates, and the results represent the means ± SD.

**Additional file 3: Investigation of DNA analogs induced activation of the IFN-β or ISRE promoter in HEK293 cells**. (A) HEK293 cells were transfected with the IFN-β or ISRE luciferase reporter plasmids and β-galactosidase or empty vectors for 24 h. After 24 h of transfection, the cells were stimulated with transfected poly(dA:dT) (1 μg/ mL) for 24 h. A luciferase assay was performed 24 h later. (B) HEK293 cells were stimulated with transfected poly(dA:dT) (1 μg/mL) at the indicated times. The cells were lysed and subjected to immunoblot analysis using the indicated antibodies. β-actin was used as a loading control. The iBright analysis software quantified the band intensities (Invitrogen, Version 5.2.0). The experiments were carried out in triplicates, and the results represent the means ± SD. One-way ANOVA with Tukey’s multiple comparisons test was used to determine the significance level. ***p < 0.001.

## Data Availability

The datasets analysed during this study are included in this published article.

## Abbreviations

ASFV: African swine fever virus
LFBK: foetal porcine kidney cell line
PAM: porcine alveolar macrophage
ISRE: interferon-stimulated response element
NF-κB: nuclear factor kappa-light-chain-enhancer of activated B cells
JAK-STAT: Janus kinase-signal transducer and activator of transcription
IFN-β: interferon-beta
IFN-α: interferon-alpha
ISG15: interferon-stimulated gene 15
ISG56: interferon-stimulated gene 56
MX1: Myxovirus resistance 1
IRF7: interferon regulatory factor 7
IRF3: interferon regulatory factor 3
PKR: protein kinase R
