# Supplementary figures and images for: Highly suitable LFBK cells for African swine fever virus replication and type I interferon-induced immune studies
Source: Vet Res. 2025 Jun 11;56:116. doi: 10.1186/s13567-025-01543-7 (PMC12153162; doi:10.1186/s13567-025-01543-7)

A

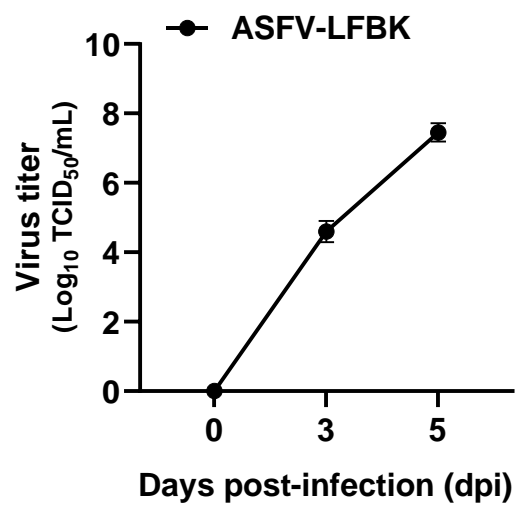

B

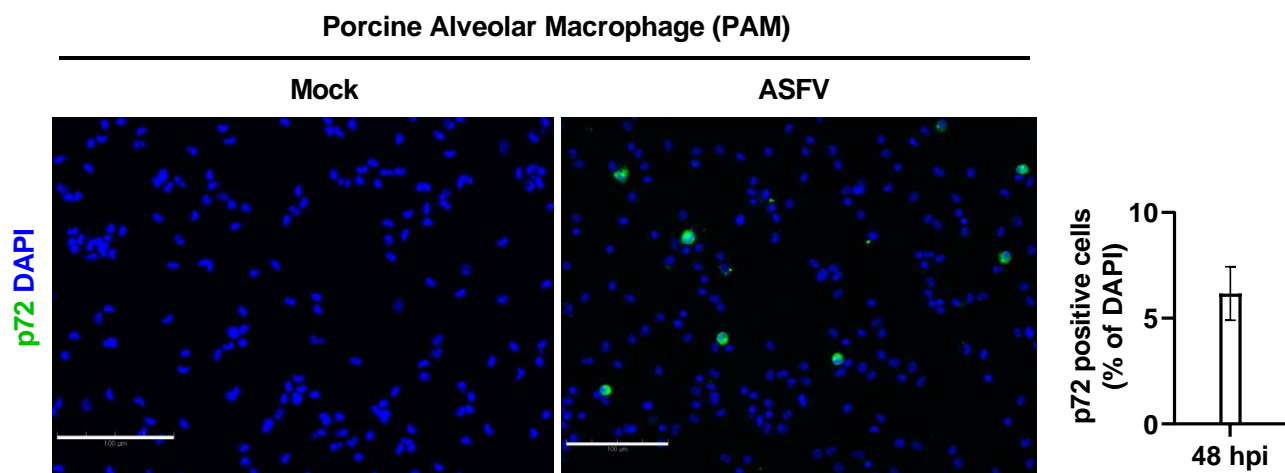

Supplement: Supplementary file 1 — Additional file 1: Determination of the ASFV infectivity in LFBK cells or PAMs. (A) The LFBK cells were infected with 10-fold serially diluted from 1 × 10^−8^ to 1 × 10^−1^ diluents of ASFV for 3 to 5 days post-infection (dpi). A 50% tissue culture infectious dose (TCID50) was determined based on whether or not a cytopathic effect occurred. The TCID50 was calculated using the Reed and Muench method. (B) PAM cells were non-infected or infected with ASFV (MOI of 0.1). Representative immunofluorescence images of the ASFV p72 protein (green) and DAPI (blue) staining in PAM cells (48 hpi). The experiments were carried out in triplicates, and the results represent the means ± SD. Magnification: 200×. [file 13567_2025_1543_MOESM1_ESM.pdf]

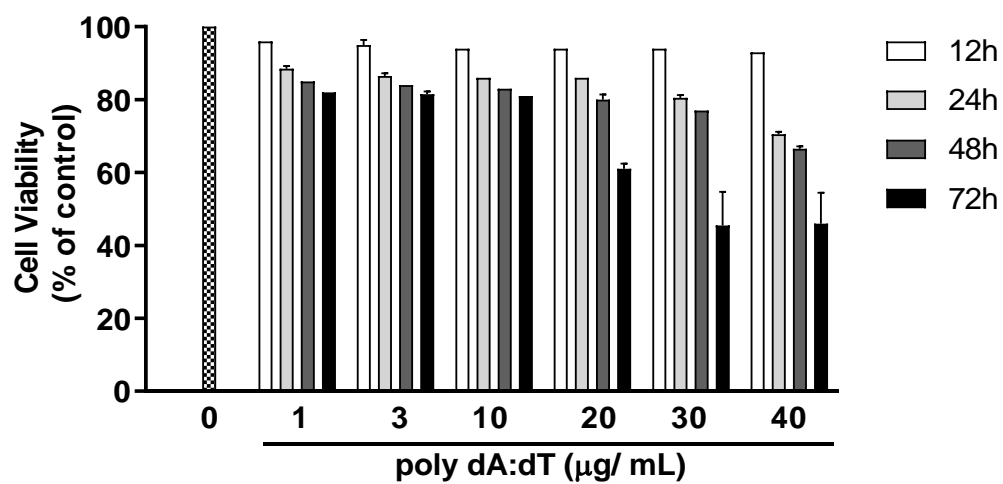

Supplement: Supplementary file 2 — Additional file 2: Cell viability analysis of LFBK cells treated with varying poly(dA:dT) concentrations. Luminescent cell viability assay was performed at subsequent times after the cells were dose-dependently stimulated with transfected poly(dA:dT) compared to the non-transfected control group. The experiments were carried out in triplicates, and the results represent the means ± SD. [file 13567_2025_1543_MOESM2_ESM.pdf]

**A**

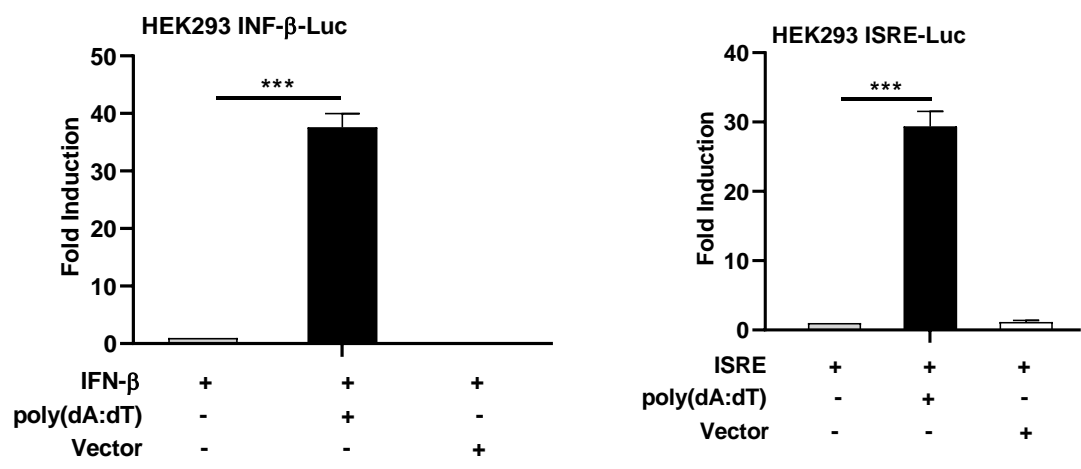

**B**

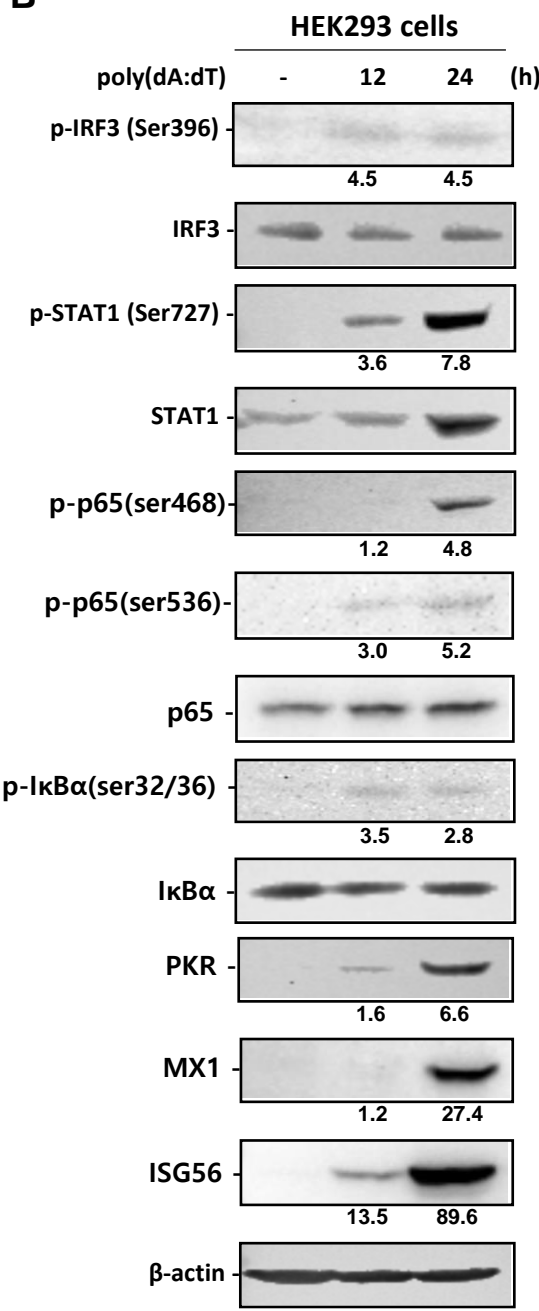

Supplement: Supplementary file 3 — Additional file 3: Investigation of DNA analogs induced activation of the IFN-β or ISRE promoter in HEK293 cells. (A) HEK293 cells were transfected with the IFN-β or ISRE luciferase reporter plasmids and β-galactosidase or empty vectors for 24 h. After 24 h of transfection, the cells were stimulated with transfected poly(dA:dT) (1 μg/ mL) for 24 h. A luciferase assay was performed 24 h later. (B) HEK293 cells were stimulated with transfected poly(dA:dT) (1 μg/mL) at the indicated times. The cells were lysed and subjected to immunoblot analysis using the indicated antibodies. β-actin was used as a loading control. The iBright analysis software quantified the band intensities (Invitrogen, Version 5.2.0). The experiments were carried out in triplicates, and the results represent the means ± SD. One-way ANOVA with Tukey’s multiple comparisons test was used to determine the significance level. ***p < 0.001. [file 13567_2025_1543_MOESM3_ESM.pdf]
